# Improving the Purity of Extracellular Vesicles by Removal of Lipoproteins from Size Exclusion Chromatography- and Ultracentrifugation-Processed Samples Using Glycosaminoglycan-Functionalized Magnetic Beads

**DOI:** 10.1021/acsami.4c03869

**Published:** 2024-08-16

**Authors:** Cheng-Yu Chou, Po-Chieh Chiang, Chih-Chi Li, Jheng-Wun Chang, Po-Han Lu, Wei-Fan Hsu, Li-Chun Chang, Jung-Lung Hsu, Ming-Shiang Wu, Andrew M. Wo

**Affiliations:** † Institute of Applied Mechanics, National Taiwan University, 1 Roosevelt Road, Sec. 4, Taipei 106319, Taiwan; ‡ Reliance Biosciences, Inc., New Taipei City 231023, Taiwan; § Graduate Institute of Biomedical Electronics and Bioinformatics, National Taiwan University, Taipei 106319, Taiwan; ∥ Department of Internal Medicine, National Taiwan University Hospital, Taipei 100225, Taiwan; ⊥ Health Management Center, National Taiwan University Hospital, Taipei 100225, Taiwan; # Department of Neurology, New Taipei Municipal TuCheng Hospital, New Taipei City 236017, Taiwan; ∇ Department of Neurology, Chang Gung Memorial Hospital Linkou Medical Center and College of Medicine, Neuroscience Research Center, Chang-Gung University, Linkou, Taoyuan 33302, Taiwan; ○ Graduate Institute of Mind, Brain, & Consciousness, Taipei Medical University, Taipei 110301, Taiwan

**Keywords:** second-step, extracellular vesicles, exosomes, lipoproteins, size exclusion chromatography, glycosaminoglycans, LipoMin

## Abstract

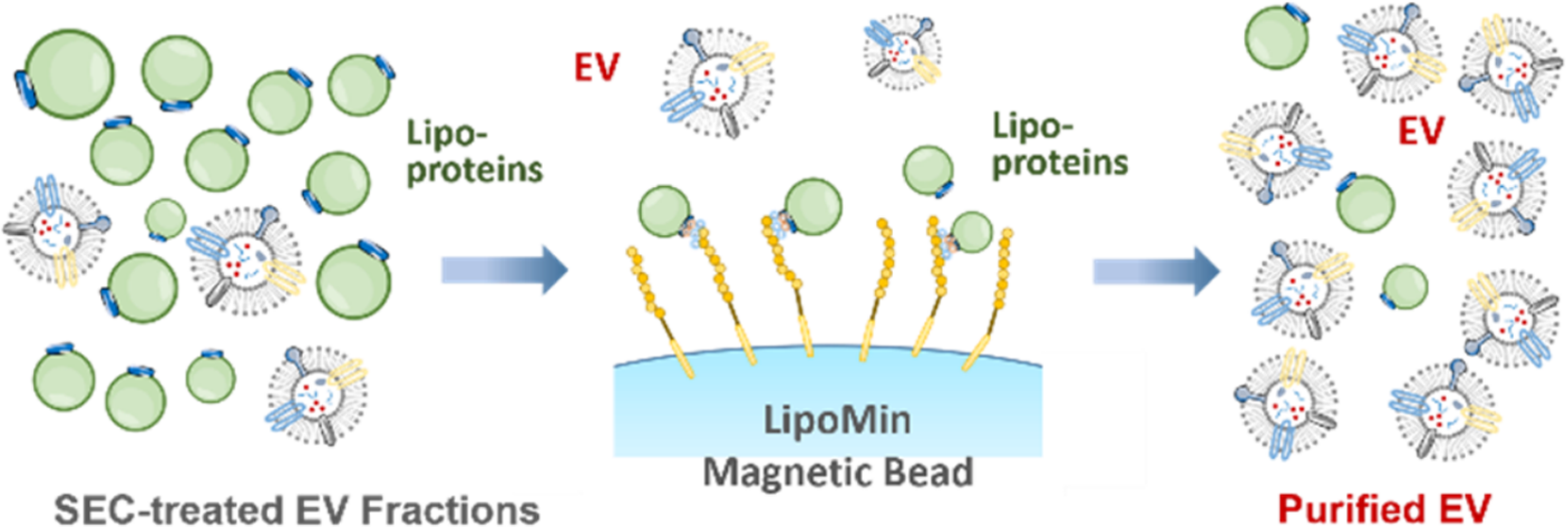

Extracellular vesicles (EVs) are present in blood at much lower concentrations (5–6 orders of magnitude) compared to lipoprotein particles (LP). Because LP and EV overlap in size and density, isolating high-purity EVs is a significant challenge. While the current two-step sequential EV isolation process using size-expression chromatography (SEC) followed by a density gradient (DG) achieves high purity, the time-consuming ultracentrifugation (UC) step in DG hinders workflow efficiency. This paper introduces an optimized magnetic bead reagent, LipoMin, functionalized with glycosaminoglycans (GAGs), as a rapid alternative for LP removal during the second-step process in about 10 minutes. We evaluated LipoMin’s efficacy on two sample types: (a) EV fractions isolated by size exclusion chromatography (SEC + LipoMin) and (b) the pellet obtained from ultracentrifugation (UC + LipoMin). The workflow is remarkably simple, involving a 10 min incubation with LipoMin followed by magnetic separation of the LP-depleted EV-containing supernatant. Results from enzyme-linked immunosorbent assay (ELISA) revealed that LipoMin removes 98.2% ApoB from SEC EV fractions, comparable to the LP removal ability of DG in the SEC + DG two-step process. Importantly, the EV yield (CD81 ELISA) remained at 93.0% and Western blot analysis confirmed that key EV markers, flotillin and CD81, were not compromised. Recombinant EV (rEV), an EV reference standard, was spiked into SEC EV fractions and recovered 89% of CD81 protein. For UC + LipoMin, ApoA1 decreased by 76.5% while retaining 90.7% of CD81. Notably, both colorectal cancer (CRC) and Alzheimer’s disease (AD) samples processed by SEC + LipoMin and UC + LipoMin displayed clear expression of relevant EV and clinical markers. With a 10 min workflow (resulting in a 96% time saving compared to the traditional method), the LipoMin reagent offers a rapid and efficient alternative to DG for LP depletion, paving the way for a streamlined SEC + LipoMin two-step EV isolation process.

## 1 Introduction

Extracellular vesicles (EVs) consist of a heterogeneous population of lipid bilayer membrane structure, roughly 30 nm to a few micrometers in diameter, and are secreted from cells as a routine physiological process. Exosomes, an important subset of EV, have their biogenesis from multivesicular endosomes and contain proteins, lipids, and nucleic acids.^1−3^ It is now well known that EVs play an important role in disease progression, immunity, and interaction with therapeutics.^4^ As such, high-purity EVs are invaluable for research and applications in a myriad of clinical indications.

**Figure 1:**
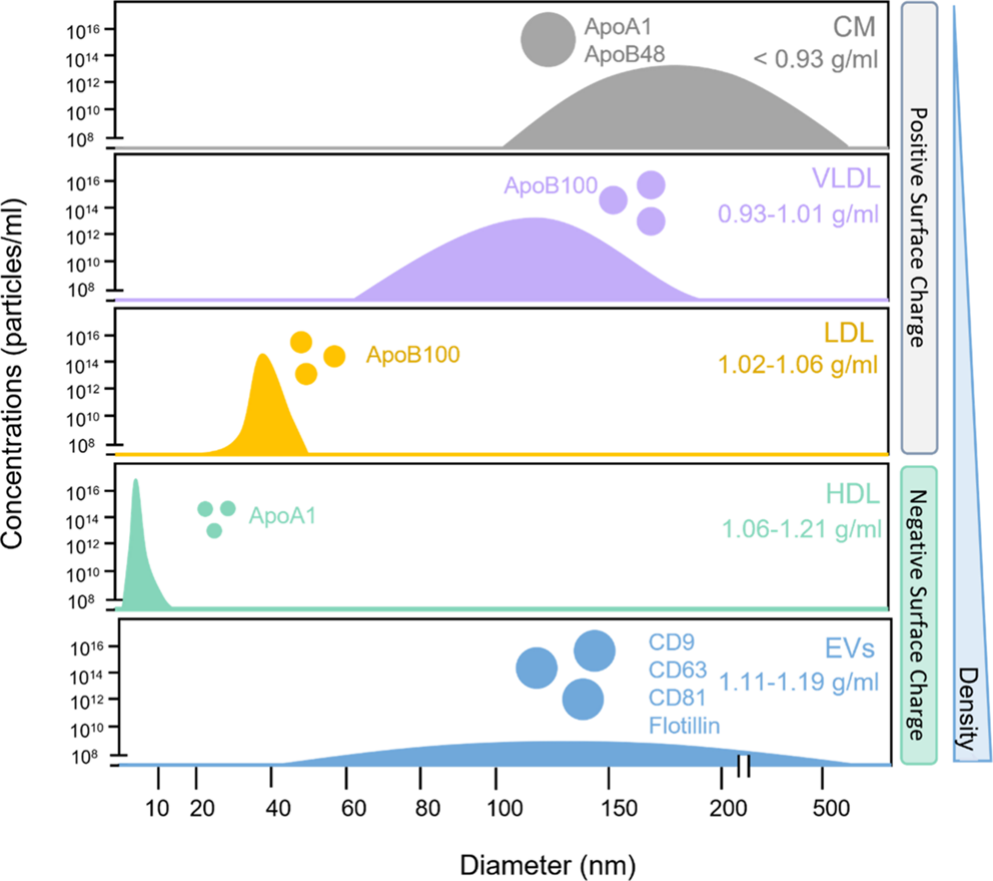
Illustration of concentration, particle size, surface charge, and density of lipoprotein particles (LP) and EV. LP are 5–6 orders of magnitude more than EV, and with much overlap in size and density, rendering high-purity EV isolation extremely challenging. Surface charge is exploited to enable rapid, high-purity EV isolation in this work.

Hindering progress toward highly pure EV from plasma or serum is the fact that there are roughly 10^16^ lipoprotein particles (LP) compared to 10^10^ EVs.^5,6^ In peripheral blood, LP include high-density LP (HDL, 5–12 nm, 1.063–1.210 g/cm^3^), low-density LP (LDL, 18–25 nm, 1.019–1.063 g/cm^3^), intermediate-density LP (IDL, 25–35 nm, 1.006–1.019 g/cm^3^), very low-density LP (VLDL, 30–80 nm, 0.930–1.006 g/cm^3^), and chylomicrons (CM, 75–1200 nm, <0.930 g/cm^3^).^7,8^ On top of the 6-order of magnitude difference between LP and EV quantities, the two populations have similar sizes and densities as shown in Figure 1.^6,9,10^ Further, some proteins bind to both lipoproteins and EVs. For instance, anti-apolipoprotein A1 (ApoA1) binds with HDL and CM. Anti-apolipoprotein B (including ApoB100 and ApoB48) binds with LDL, VLDL, and CM.^8,9^ Fortunately, CD9, CD63, CD81, and flotillin bind somewhat distinctively to EVs and are commonly recognized as markers of EVs.^11^

Despite paramount challenges, much progress has been made toward the isolation of EV from plasma. For many years, one-step isolation method, e.g., differential centrifugation,^12,13^ and size exclusion chromatography (SEC)^14^ have been well accepted but not without challenges. For example, stand-alone SEC process co-isolates EV with VLDL and chylomicrons (CM) due to their similar sizes.^15,16^ Further, stand-alone UC isolates EV with much HDL due to similar densities.^17,18^ Some polymer-based methods, e.g., ExoQuick, can result in increased viscosity of the sample mixture, leading to uncertainties in repeatability during the pipetting process and might also co-precipitate contaminants along with target EVs.^12,19,20^ Understanding of lipoprotein characteristics have also been leveraged to remove LP, e.g., antigen–antibody binding,^21,22^ lipoprotein–ligand adsorbing,^22,23^ electrical property,^24,25^ and pH regulation.^26,27^ Further, several studies utilized difference in the electrical properties of EV and LP.^28,29^

Surface charge properties were also been interrogated. Because the phosphatidylserine on the surface of EV is negatively charged, EV are believed to be negatively charged, as shown in Figure 1.^30^ Other studies have found that LDL can bind to the negatively charged SO_4_^–^ groups on the GAG due to positively charged ApoB, where LDL and VLDL are also positively charged as shown in Figure 1.^23,31^ At pH 7.4, the ζ potential results also showed that LDL and VLDL were positively charged relative to EVs.^28,29^ Thus, this difference in electrical properties between EV and LP might enable the removal of LP, particularly those that are positively charged.

The quest for high-purity EV for some applications, e.g., proteomic studies, thrusts further advancement of EV isolation methods.^32−34^ Since high-purity EV cannot be solely accomplished by a single-step purification, two-step isolation approach has currently been widely used.^16,35−37^ The rationale here is to sequentially process plasma (or serum) samples based on two properties to untangle the mixed EV and LP populations in density and size. For example, SEC is often first used followed by density gradient (DG) approach.^35^ This two-step process is necessary to remove considerable LP contamination in EV fractions in the post-SEC sample. Another method based on the size and density of particles is the combination of three sequential isolation methods including UC, density cushion, and SEC.^7,20^ In addition, cation exchange has also been applied in the dual-mode chromatography (DMC) to improve the SEC process.^6,38^ DMC consists of a top layer of 10 mL of sepharose and a bottom layer of 2 mL of fractogel. Due to the negative charge of fractogel particles, it can attract positively charged LDL and VLDL. Meanwhile, negatively charged EVs are not affected and can continue to flow downstream. Compared to the stand-alone SEC process, the DMC process does not increase much experimental time. It can also exclude VLDL that is similar to EVs in particle size. Although much progress has been made, removal of LP from plasma remains a niche process and is challenging to transform to a rapid and routine laboratory workflow. Even though there are commercially available reagents for lipoprotein removal,^39^ they are still not widely accepted for a range of reasons, e.g., efficacy and cost.

This paper presents a novel glycan-based reagent (LipoMin) for the removal of LP from UC pellet and SEC-treated EV fractions both within 10 min of processing. LipoMin reagent contains magnetic beads functionalized with glycosaminoglycans (GAG) polymer.^40^ LipoMin is designed as the second step in the two-step EV isolation process and rapidly removes LP from the sample processed by UC or SEC as the first step. LipoMin has the potential to replace DG for SEC-treated fractions, enabling a much faster workflow. Interrogation of the efficacy of LipoMin includes NTA, transmission electron microscopy (TEM), western blot (WB), and sandwich enzyme-linked immunosorbent assay (ELISA) of ApoA1, ApoB, and exosomal proteins of pre- and post-LipoMin processing. Plasma samples from colorectal cancer (CRC) and Alzheimer’s disease (AD) patients were also tested with LipoMin for its utility on clinical samples.

## 2 Experimental Section

### 2.1 Blood Sample Preparation

Peripheral blood was collected using ethylenediaminetetraacetic acid (EDTA) tubes from healthy donors, colorectal cancer (CRC), and Alzheimer’s disease (AD) patients, which was approved by the IRB of the National Taiwan University Hospital and the Chang Gong Memorial Hospital. Blood samples were centrifuged at 1500*g* for 15 min under 4 °C to remove the formed elements. Then, the supernatant of the plasma was isolated by centrifugation at 12,000*g* for 30 min under 4 °C to remove the cell debris and the residual platelets. The platelet-depleted blood plasma was collected and stored at −80 °C.

### 2.2 Size Exclusion Chromatography (SEC)

SEC was conducted according to the previous published methods.^35,41^ Sepharose CL-2B (GE Healthcare, Uppsala) was used for the SEC column to treat the plasma sample as the first purification step. The SEC column was prepared by placing a nylon net with 25 μm pore size (Tricorn Filter Kits, coarse, Cytiva) at the bottom of a 10 mL column (Empty Disposable PD-10 Columns, Cytiva), followed by packing of 10 mL of Sepharose CL-2B per the manufacturer’s instruction. (Note that the volume of 10 mL of Sepharose gel after packing is actually less than 10 mL.) Prior to loading the sample, the SEC column requires complete liquid exchange by washing with 30 mL of phosphate-buffered saline (PBS) buffer over 10 times, with 3 mL added slowly each time. During wash, the outlet of column is open, discharging fluid and enabling liquid exchange between PBL and Sepharose gel. 2 mL of plasma was slowly loaded into the column (1–2.5 mL plasma can be added per manufacturer’s instruction). Afterward, PBS was dripped slowly into the column while simultaneously collecting 16 sequential 1 mL eluted fractions (1 mL per fraction). Alternatively, 4 mL of plasma was also fractionated using two parallel SEC columns, and the corresponding fractions of the two columns were pooled together. The SEC fractions F3 to F5, identified as EV-containing fractions per CD81 signal (see Figure 4b), were pooled and then processed by one of the two second-step methods, i.e., LipoMin reagent or by density gradient treatment (as comparison).

### 2.3 Ultracentrifugation (UC)

1 mL of blood plasma was loaded into a centrifuge tube and was centrifuged at 100,000*g* for 2 h (Optima MAX-XP, Beckman Coulter, CA) to treat the plasma sample as the first-step EV isolation process. Then, the pellet was resuspended using 1 mL of PBS buffer (pH 7.4, GOAL Bio, Taiwan, 0.22 μm filtered) and recentrifuged at 100,000*g* for another 2 h. Finally, the pellet was resuspended using 1 mL of PBS buffer. It is mixed with LipoMin in the second-step process.

### 2.4 Sucrose Density Gradient (DG)

Sucrose (Sigma-Aldrich) solutions 2.5, 1.3, 0.95, 0.6, 0.3, and 0.15 M were prepared in 250 mM Tris (J.T. Baker) buffer. A discontinuous sucrose gradient was prepared by layering 2.5, 1.3, 0.95, 0.6, 0.3, and 0.15 M sucrose slowly in a polyamide tube (Himac-science, Japan). The diluted SEC EV-containing fraction sample (1 mL) was put on the top of the tube gently without mixing the layers and then ultracentrifugation at 100,000*g* and 4 °C for 17.5 h using a P40ST swinging bucket rotor (Hitachi, Japan). After centrifugation, ten 1 mL fractions were collected starting from the top of the tube and stored at −20 °C for downstream applications.

### 2.5 LipoMin Reagent

**Figure 2:**
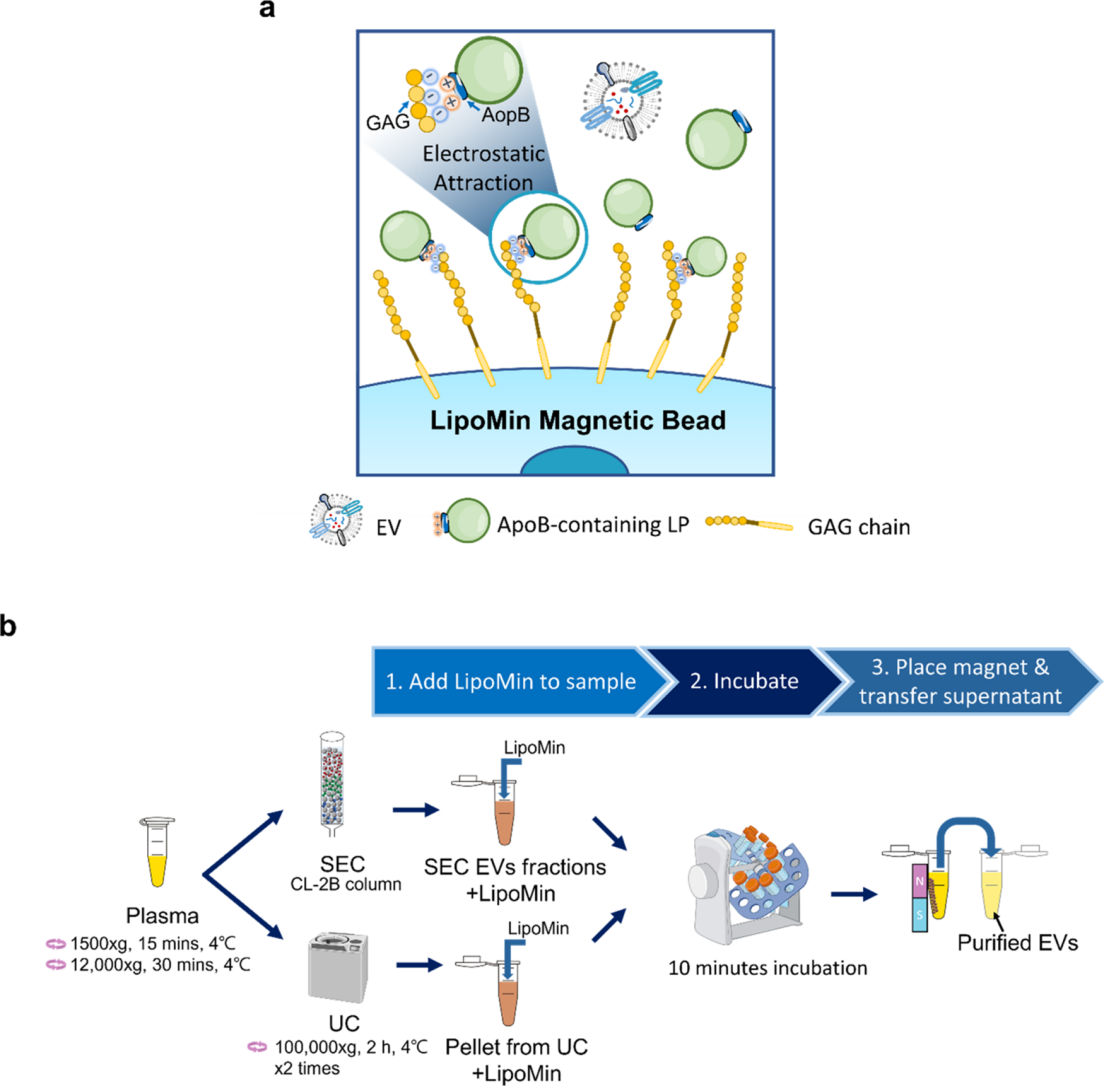
(a) Illustration of the mechanism by which LipoMin captures LP where electrostatic attraction enables LP to attract to LipoMin magnetic beads. (b) Graphical workflow of the LipoMin process: (1) mixing the sample with LipoMin, (2) incubating for 10 min, and (3) placing a magnet near the incubated sample and transferring the supernatant containing purified EV.

LipoMin reagent (product# RB02001, Reliance Biosciences Inc., Taiwan) was used to further remove LP from the UC pellet and SEC EV pooled fractions, enabling extremely pure EVs. The reagent contains functionalized magnetic beads that are coupled with the negatively charged glycosaminoglycans (GAG). This GAG substance has a carboxylate structure. The use of electrostatic attraction between glycan and positively charged LP (Figure 2a) is more cost-effective than using antibody affinity for LP removal. The protocol provided by the manufacturer was followed to process samples using the LipoMin reagent. Further details in the preparation of LipoMin and dilution ratios are provided in the Supporting Information.

Figure 2b shows the workflow of LipoMin which is extremely straightforward. The sample (SEC EV fractions or UC pellet) was mixed with LipoMin reagent using the suggested volume according to the manufacturer’s instructions. After mixing well by pipetting, the mixture was incubated on the rotator for 10 min. Then, a magnet stand was used to remove LipoMin magnetic beads capturing LP from the mixture. Supernatant containing purified EV was transferred to a new container for downstream processing.

### 2.6 Antibodies

Experimental details including antibodies using enzyme-linked immunosorbent assay and western blot in this study are summarized in Table S1 (Supporting Information).

### 2.7 Recombinant EV (rEV)

In an important milestone in standardization and comparison of EV data, recombinant EV (rEV) with fluorescence was generated as a biological reference material by Prof. Hendrix’s group.^42^ Thus, spiking rEV in preprocessed samples enables direct comparison of efficiency of EV recovery for various EV isolation methods. In this work, lyophilized rEV (product SAE0193, Sigma-Aldrich)—believed to be the commercialized product of the paper and Prof. Hendrix cited this in her ISEV2023 Education Day talk—was used. The entire stock of lyophilized rEV was reconstituted with 100 μL of DI water per the manufacturer’s instruction. NTA result (SS signal) showed that the rEV concentration of this reconstituted stock was 7.96 × 10^9^ particles/mL or 7.96 × 10^8^ particles per vial (with 100 μL DI water), not the greater than 1 × 10^9^ particles per vial stated in the product COA. Further interrogation of rEV fluorescent signal showed an NTA SS signal of 3.43 × 10^9^ particles/mL resulted in an NTA fluorescent signal (488 nm excitation) of 1.39 × 10^9^ particles/mL, hence 40.5% of rEV possess fluorescence (datasheet says ≥70%). Throughout this work, CD81 sandwich ELISA was used for all rEV-related quantification.

Then, rEV was used to enable quantification of the efficiency of EV recovery using LipoMin in the SEC + LipoMin process. First, 2.8 μL of reconstituted rEV or 2.21 × 10^7^ rEV particles was quantified with CD81. Second, 5 μL of SEC F3–F5 fraction pooled sample (see Section 2.2) was also quantified. Third, 5 μL of SEC F3–F5 fractions pooled sample was spiked with 2.21 × 10^7^ rEV particles, and CD81 of the mixture was measured. Fourth, CD81 of 5 μL of the SEC portion with LipoMin was also quantified. Fifth, a mixture of 5 μL of SEC F3–F5 fractions pooled sample spiked with 2.21 × 10^7^ rEV was processed with LipoMin, and CD81 was quantified. Lastly, recoveries of CD81 pre- and post-LipoMin for the samples stated were calculated, and results are presented in Figure 5 and discussed in Section 3.2.

### 2.8 Protein Quantitation Assay

The protein concentration of samples was measured using BCA protein assay (Pierce BCA Protein Assay Kit, Thermo Fisher), according to the manufacturer’s instructions. UC pellet and SEC EV fractions were diluted in different ratios, respectively, and the final volume was adjusted to 25 μL. Then, the diluted sample was mixed with the working reagent and loaded into the opaque plate (Greiner Bio-one, Germany). After incubation for 30 min at 37 °C, the microplate reader (SpectraMax iD3Microplate Reader, Molecular Devices) measured absorbance at 526 nm.

### 2.9 Enzyme-Linked Immunosorbent Assay (ELISA)

Quantification of ApoA1, ApoB, and CD81 proteins was performed using an in-house chemiluminescent ELISA system. Recombinant proteins of ApoA1 (Abcam, U.K.), ApoB (Abcam, U.K.), and CD81 (MyBioSource) were used in the sandwich ELISA. Protein concentrations for ApoA1, ApoB, and CD81 were calculated by correlating the intensity obtained from the microplate reader (SpectraMax iD3Microplate Reader, Molecular Devices) with the concentration of recombinant proteins.

### 2.10 Western Blot (WB)

Samples were diluted to a uniform protein concentration (15 μg of protein per sample) and mixed with sample buffer (4× Laemmli sample buffer/2-mercaptoethanol = 9:1, Bio-Rad, Benicia, CA) at a ratio of 3:1 to load in each gel land. Then, 95 °C heat was applied for 15 min. Proteins were separated by gel electrophoresis (Mini-PROTEAN TGX Gels, 4–15%, Bio-Rad) and transferred to immobilon membranes (Immobilon-P Transfer Membranes, pore size 0.45 μm Merck Lifescience, Germany). After blocking the membranes with 5% bovine serum albumin (BSA, Alpha Biochemistry, Taiwan) in PBS with 0.05% Tween 20 (Sigma-Aldrich) for 1.5 h, the blots were incubated overnight with primary antibodies. One hour incubation with secondary antibodies was performed after extensive washing of the membranes in PBS with 0.5% Tween 20. After final extensive washing, chemiluminescence substrate (SuperSignal ELISA femto substate, Thermo Fisher) was added, imaging was performed using the UVP (UVP ChemStudio PLUS Touch, Analytik Jena AG, Germany), and the images were analyzed (VisionWorks and ImageJ software).

### 2.11 Nanoparticle Tracking Analysis (NTA)

EV size distributions were analyzed by NTA Instruments (NanoSight NS300, Malvern Panalytical, U.K.). For each analysis, three videos of 60 s were recorded and analyzed with a camera level of 16 and a detection threshold of 5. The temperature was monitored during the recording. Recorded videos were analyzed with NTA software. For optimal measurements, the samples were diluted with PBS until particle concentration was within the concentration range for the NTA software (10^6^–10^9^ particles/mL).

### 2.12 Transmission Electron Microscopy (TEM)

TEM was used to observe the morphology of the EVs at each plasma treatment stage. Samples were diluted in 0.1 M phosphate buffer (Sigma-Aldrich) and deposited on a carbon film 200 mesh copper (Electron Microscopy Sciences). Then, the samples were fixed with 1% glutaraldehyde and incubated for 5 min. The meshes were washed with phosphate buffer and DI water and then stained with 2% uranyl acetate. Prepared meshes were examined (H-7650 TEM, Hitachi, Japan), and the images were captured at 120 kV.

### 2.13 Data Sampling and Analyses

**Figure 3:**
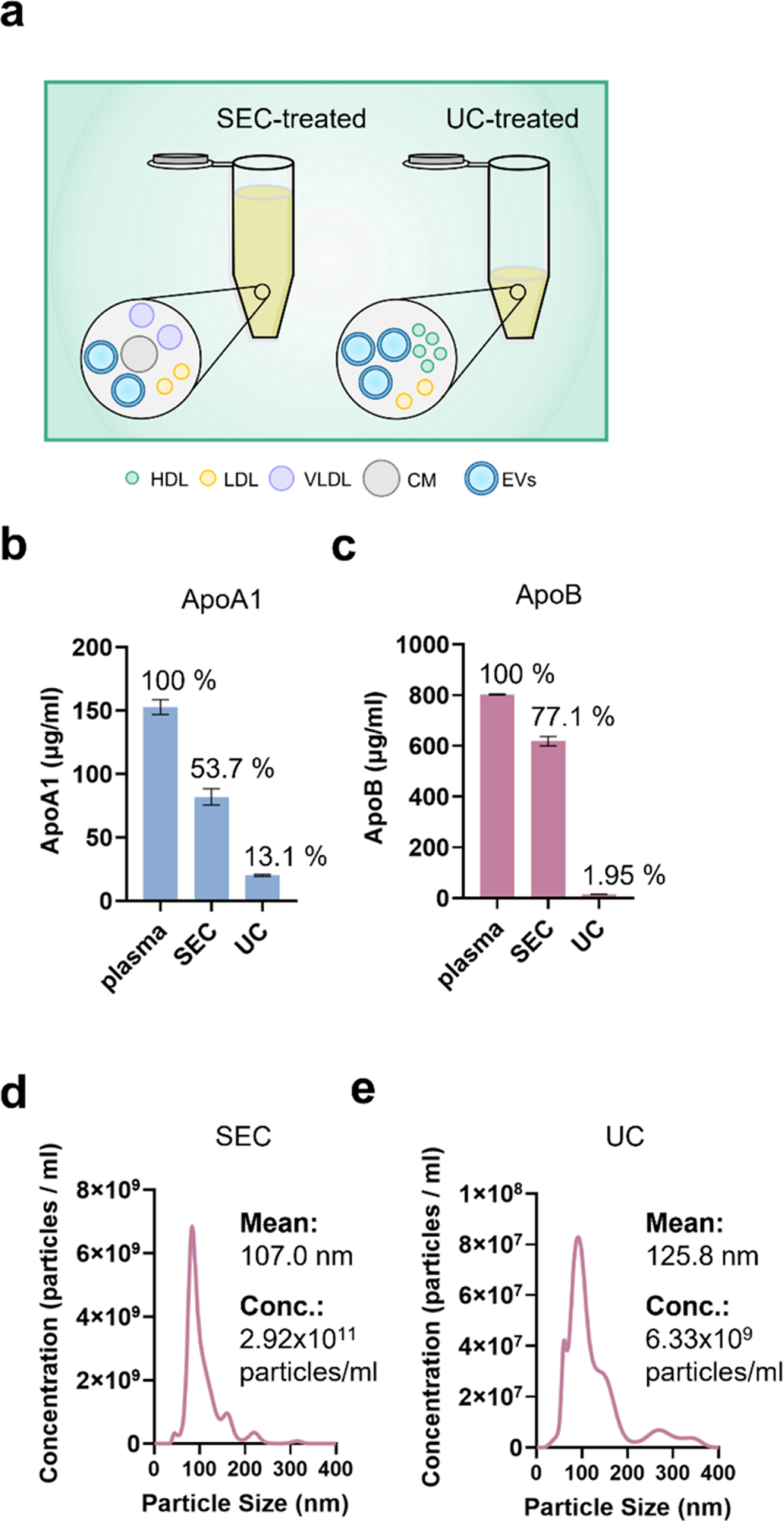
Characteristics of plasma samples processed by SEC-only and UC-only, both without LipoMin. (a) Illustration of SEC-only and UC-only both co-isolated EV with LP. LP population is dominated by CM and LDL after SEC, and HDL after UC. Sandwich ELISA analysis of (b) ApoA1 and (c) ApoB for SEC (pooled F3–F5 EV fractions, see Figure 4b,c) and UC (pellet resuspended, see Figure 5). NTA results for (d) SEC (pooled F3–F5 EV fractions) and (e) UC (pellet resuspended). ELISA data were from two replicates after repeated testing at least four times to ensure consistency. Error bars represent standard deviations. NTA was performed twice.

**Figure 4:**
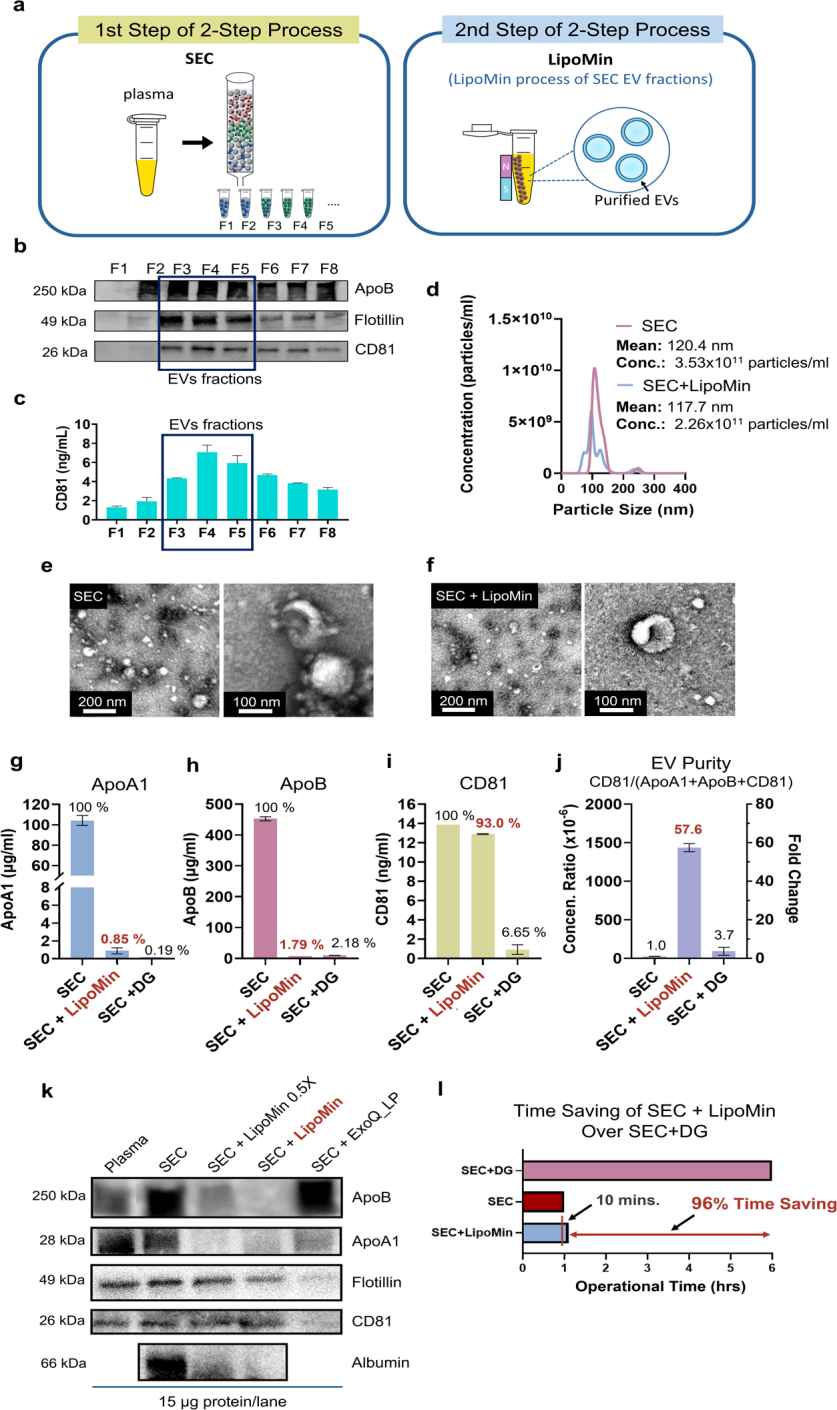
Size exclusion chromatography (SEC) followed by LipoMin (SEC + LipoMin) to enable high-purity EV with over 90% time savings. (a) LipoMin workflow: (1) process plasma sample with SEC to obtain EV fractions, (2) mix LipoMin with EV fraction via pipetting, place mixture under magnetic stand to capture LP on LipoMin magnetic beads, and then aspirate supernatant containing purified EV. (b) Western blot analysis (ApoB, CD81, and flotillin) of EV fractions and (c) sandwich ELISA analysis (CD81) of the corresponding EV fractions. (d) NTA of SEC-only and SEC + LipoMin processed samples. (e) TEM images (wide field and high resolution) of SEC-only and (f) SEC + LipoMin processed samples. (g) Sandwich ELISA analyses of ApoA1, (h) ApoB, and (i) CD81 for SEC-only, SEC + LipoMin, and SEC + DG. (j) Calculated EV purity, defined as CD81/(ApoA1+ApoB+CD81), based on ELISA data of (g)-(i). (k) WB of ApoB, ApoA1, flotillin, CD81, and albumin for SEC-only, SEC + LipoMin 0.5× (half the volume of LipoMin but at the same concentration), SEC + LipoMin, and ExoQuick_LP (stated as ExoQ_LP). (l) Comparison of workflow duration among SEC + DG, SEC-only, and SEC + LipoMin reveals over 96% time saving is attained using LipoMin over that of DG as a post-SEC 2nd step process. ELISA data were from two replicates after repeated testing at least four times to ensure consistency. Error bars represent standard deviations. WB and NTA were performed twice.

**Figure 5:**
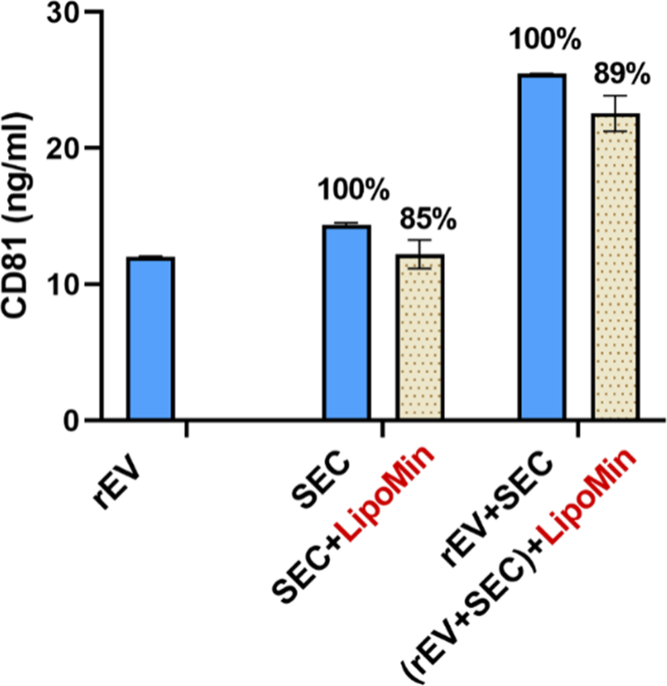
Recombinant EV (rEV) was used as the standard material to quantify the efficiency of recovery of EV in SEC pooled fractions processed with LipoMin (SEC + LipoMin). The following material were quantified with CD81 sandwich ELISA: rEV (reconstituted from lyophilized material) of 2.21 × 10^7^ particles (labeled as “rEV”)—resulted in CD81 of 12.0 ng/mL; 5 μL of SEC F3–F5 pooled fractions (“SEC”)—14.5 ng/mL; 5 μL of SEC pooled fractions spiked with 2.21 × 10^7^ rEV (“rEV + SEC”)—25.5 ng/mL; 5 μL of SEC pooled fractions processed with LipoMin (“SEC + LipoMin”)—12.2 ng/mL; 5 μL of SEC pooled fractions spiked with 2.21 × 10^7^ rEV and the mixture processed with LipoMin (“(rEV + SEC) + LipoMin”)—22.6 ng/mL. The recovery was 85% (=12.2/14.5) for SEC + LipoMin compared to SEC baseline, and 89% (=22.6/25.5) for the use of LipoMin to retain substantial CD81 protein. Data were from two replicates after repeated testing at least four times to ensure consistency. Error bars represent standard deviations.

**Figure 6:**
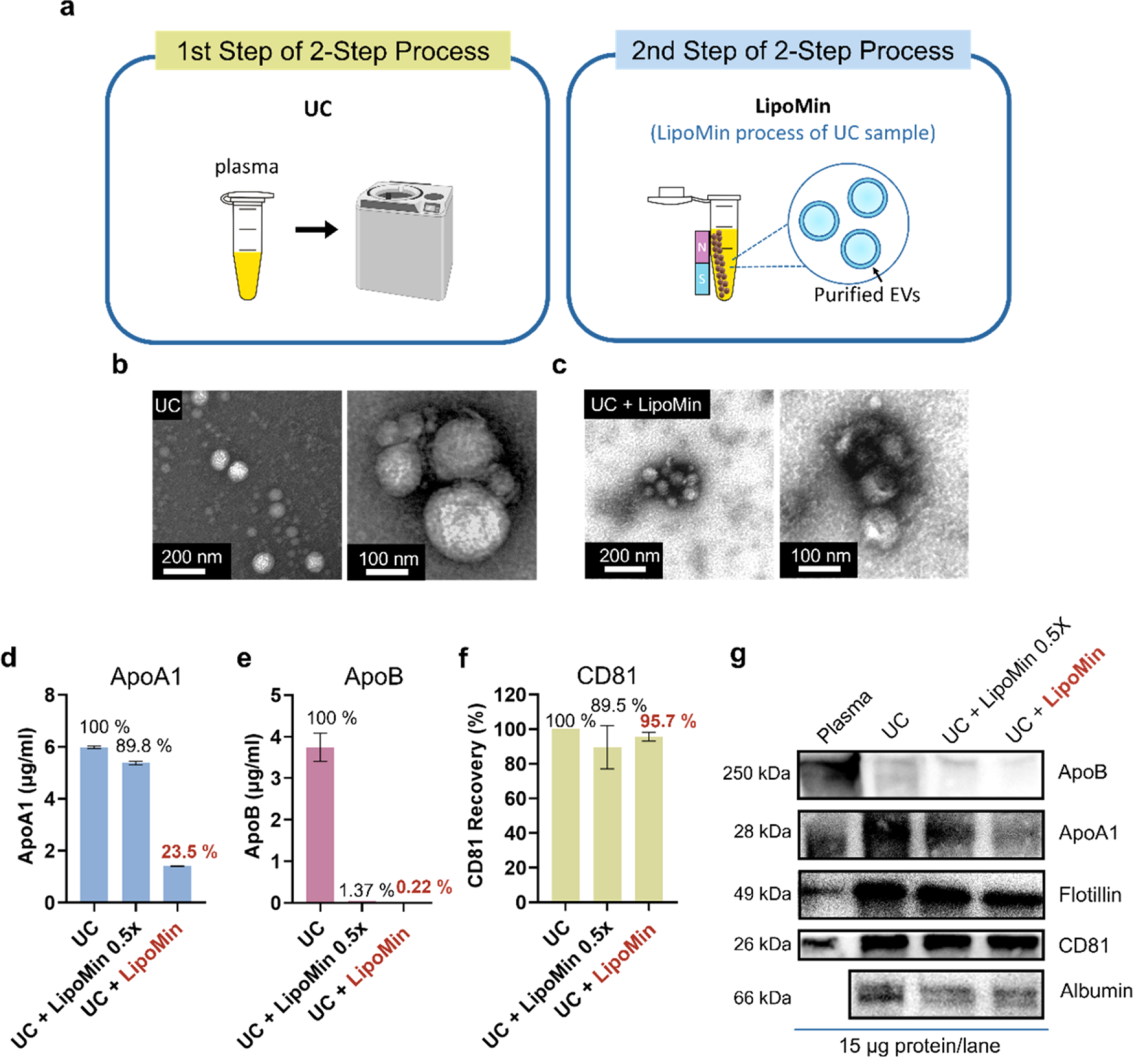
Characteristics of differential ultracentrifugation (UC) followed by LipoMin (UC + LipoMin) process to isolate EV from plasma. (a) Two-step workflow: first step: UC, second step: resuspend UC pellet, add LipoMin reagent, mix gently via pipetting, place magnet stand next to Eppendorf, pipette out sample containing purified EV. (b) TEM images of resuspended UC pellet. (c) TEM images of UC + LipoMin sample. Sandwich ELISA analysis of (d) ApoA1, (e) ApoB, and (f) CD81. (g) Western blot of ApoB, ApoA1, flotillin, CD81, and albumin for UC pellet, UC + LipoMin 0.5× (half-volume of standard LipoMin reagent but at the same concentration), and UC + LipoMin. ELISA data were from two replicates after repeated testing at least four times to ensure consistency. Error bars represent standard deviations. WB was performed twice.

Whenever feasible, each test was repeated at least four times or more, with each test consisting of two replicates, hence totaling eight or more times tested. Results of the tests showed considerable consistency, with coefficient of variation (CV) added when appropriate which includes Figures 3–6.

Data analysis and graphical presentations were performed using GraphPad Prism version 9 (GraphPad Software). Generally, bar graphs were presented by normalized to untreated sample as 100% or as the mean with the standard error. WB images were inverted to black and white images to measure the intensity of the band using ImageJ (NIH, Maryland).

### 2.14 Adherence to MISEV 2018 Guidelines

MISEV 2018 guideline^43^ on single-vesicle analysis (section 4-c) was adhered to. For section (i) on visualization, both wide-field and high-resolution TEM of single EVs were imaged (see Figures 4e,f and 5b,c). For, section (ii) on single-particle analysis (tails that do not provide high-resolution images but calculate biophysical parameters), single EVs were adhered by NTA and WB (see Figures 3d,e and 4d for NTA data and Figures 4b,j, 5g, and 6a–d for WB).

## 3 Results

### 3.1 SEC- and UC-Processed Plasma Both Co-Isolated EV with LP

Figure 3a illustrates SEC- and UC-only approaches both co-isolated EV and LP from plasma. The SEC-only process would result in predominantly LDL, CM, and EVs due to their similar size, while the UC-only approach would co-precipitate HDL with EVs due to their similar density. Figure 3b,c presents the results of sandwich ELISA from SEC-only and UC-only samples, respectively. Figure 3b shows that the LP marker ApoA1 expression decreased about 46.3% after SEC, and 86.9% after UC. This fact agrees with the understanding that ApoA1, being denser than ApoB, would be pulled down by UC more so than ApoB.^44^ For ApoB, Figure 3c shows the expression decreased 22.9% after SEC, and 98.0% after UC. Hence, a substantial amount of LP still remained, particularly ApoB after SEC.^35^

Figure 3d,e presents the NTA results of SEC- and UC-processed plasma samples, respectively. The particle concentration for SEC was 2.92 × 10^11^ particles/mL, and for UC, it was 6.33 × 10^9^ particles/mL. The substantial difference in particle concentration has been well documented and likely due to either the UC-treatment causing ruptured EV or the UC-treated sample removing a significant number of low-density lipoproteins from the plasma, resulting in a lower particle count compared to the SEC-treated sample.^10,45^ For both processes, the particle size falls within the range of EV, i.e., between 50 and 150 nm.

### 3.2 SEC + LipoMin: LipoMin Added to SEC-Processed EV Fractions

Figure 4 presents the results of SEC-processed EV pooled fractions followed by LipoMin (SEC + LipoMin) toward rapid removal of LP. Figure 4a displays the SEC + LipoMin workflow which only involves: (1) SEC process and (2) LipoMin process: mix SEC EV fractions with LipoMin reagent (contains magnetic beads) via gentle pipetting, place the mixed sample onto a magnet stand, and transfer the supernatant containing purified EV into a new container (also see Figure 2b). Figure 4b shows WB results. Both flotillin and CD81 expressions over the range of fractions are in agreement, indicating that EV should be mostly contained in fractions 3 (F3) to 5 (F5). This is substantially consistent with results from ELISA, in Figure 4c, that F3–F5 contains most of the CD81 expressions. Unequivocal identification, as much as feasible, of appropriate SEC fractions containing EV is critical. Although EV is mostly contained in F3–F5, Figure 4b shows that ApoB in these fractions remains a significant source of contamination. Thus, removal of ApoB-containing particles is important to enable high-purity EV. Toward this end, LipoMin reagent was used as a second-step purification on fractions F3–F5. Figure 4d presents the NTA results comparing SEC and SEC + LipoMin pooled fractions (F3–F5).

Figure 4e,f presents TEM wide-field and high-resolution images (per MISEV guideline) for SEC-only and SEC + LipoMin processes, respectively. The wide-field images show the characteristic population of EV, whereas high-resolution images show the cup-shaped EV morphology.

Figure 4g–i presents sandwich ELISA on SEC F3–F5 pooled fractions for ApoA1, ApoB, and CD81, respectively. Figure 4g shows that the ApoA1 expression for the SEC + LipoMin process decreased to 0.85% of the SEC-only process. Figure 4h presents data for ApoB showing the ApoB expression for the SEC + LipoMin process decreased to 1.79% of the SEC-only process. Importantly, the amount of remaining ApoA1 and ApoB after SEC + LipoMin is comparable to that of the SEC + DG process, 0.19% for ApoA1 and 2.18% for ApoB. It is also important to balance the benefit of LP decrease with its (negative) effect on EV. Data in Figure 4i shows that SEC + LipoMin retained a very strong CD81 signal (93.0%) compared to the SEC-only process (100%). This indicates that the LipoMin reagent does not seem to substantially remove EV along with LP, enabling the SEC + LipoMin process to achieve a 93% EV recovery. In contrast, the CD81 signal for the SEC + DG process was only 6.65%. This could be due to the DG process dispersing EV into other fractions, resulting in fewer EVs when selecting the EV-containing fraction (F8 and F9) in the SEC + DG process (Figure S2 in the Supporting Information).

Figure 4j presents data on EV purity, defined as the ratio of CD81 protein concentration to the sum of ApoA1, ApoB, and CD81 concentrations, based on the ELISA data in Figure 4g–i. For ease of interpretation, the numeric above a data bar states the fold change (eq 1) in EV purity with reference to SEC-only EV purity (see the right ordinate). That is
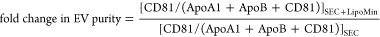
1The concentration ratio was 2.52 × 10^–5^ for SEC, 1.45 × 10^–3^ for SEC + LipoMin, and 9.30 × 10^–5^ for SEC + DG, recalling that the amount of LP is roughly 6 orders more than that of EV.^5^ These ratios resulted in the fold change in EV purity for SEC + LipoMin of 57.60, and for SEC + DG 3.69. Although 3.69 (or 369%) is substantial for SEC + DG, a 57.60-fold change is considered extremely lucrative.

Figure 4k compares WB results for ApoA1, ApoB, flotillin, CD81, and albumin with various post-SEC processes. Two LipoMin concentrations were tested: half-volume (LipoMin 0.5×) and regular volume (LipoMin), with both “LipoMin 0.5×” and “LipoMin” at the same concentration. Another commercial LP preclearing kit, ExoQuick_LP (stated as ExoQ_LP) was tested as well (see the Supporting Information for workflow details). Results show that SEC + LipoMin 0.5× substantially reduced ApoB from SEC pooled fractions (F3–F5). Further reduction in ApoB can be achieved with LipoMin. This is critical since ApoB is the single most important contaminant after SEC. ApoA1 data show both LipoMin and ExoQ_LP substantially decrease the protein from SEC-only process. Importantly, flotillin and CD81 data confirmed that they are significantly expressed even after SEC + LipoMin process. These WB data are in general agreement with the ELISA data of Figure 4g–i for ApoA1, ApoB, and CD81. Upon closer examination, when an increased amount of LipoMin is used (LipoMin vs LipoMin 0.5×), a slight decrease in flotillin and CD81 can be observed from WB results. This suggests the possibility that some EVs are removed perhaps due to their attachment to LP, or the so-called LP-EV complex.^46−48^ However, it is evident that an appropriate amount of LipoMin effectively removes lipoproteins, while strong EV signals were retained. Data also included albumin, a major source of soluble proteins in plasma. Surprisingly, LipoMin seems to have the ability to remove albumin, although this was not the intended target in the design of LipoMin. The reason for albumin reduction due to LipoMin warrants further investigation. For the SEC + ExoQ_LP process, ApoB seems not to have reduced much, while flotillin and CD81 expressions appeared to have been comprised.

A reviewer pointed out that Figure 4k seems to show a lack of enrichment of flotillin and CD81 in SEC, as compared to plasma. WB for flotillin and CD81 after SEC do seem to have a slight but noticeable enrichment over that of plasma. It should also be noted that SEC data were only from F3–F5 (pooled) with other fractions excluded, while plasma data were unpurified hence essentially from all fractions.

Figure 4l illustrates the time saving of the SEC + LipoMin process over that of SEC + DG. For the sake of this illustration, it is assumed that 1 h for SEC and 5 h for DG (under ultracentrifugation) are required, although a longer duration for DG is usually needed. With an extremely short 10 min processing time for LipoMin, SEC + LipoMin would result in workflow time saving of over 96% (=290 min/300 min) compared to the SEC + DG process. In one study,^35^ 18 h DG was used, which would result in 99.1% of time saved.

Figure 5 presents data on the use of standardized recombinant EV (rEV)^42^ to interrogate the efficiency of EV recovery using LipoMin reagent. All samples were quantified with CD81 sandwich ELISA. Several samples were tested, including baseline samples (rEV and SEC pooled fractions), spiked with rEV, and samples processed with LipoMin. Figure 5 shows (a) rEV (labeled as “rEV” in figure) resulted in CD81 protein of 12.0 ng/mL, (b) 5 μL of SEC F3–F5 pooled fractions (“SEC”)—14.5 ng/mL, (c) 5 μL of SEC pooled fractions spiked with rEVs (“rEV + SEC”)—25.5 ng/mL, (d) 5 μL of SEC pooled fractions processed with LipoMin (“SEC + LipoMin”)—12.2 ng/mL, and (e) 5 μL of SEC pooled fractions spiked with rEVs followed by the mixture processed with LipoMin (“(rEV + SEC) + LipoMin”)—22.6 ng/mL. The recovery was 85% for SEC + LipoMin compared to SEC baseline, and 89% for (rEV + SEC) + LipoMin compared to (rEV + SEC) baseline. Data, with and without rEV, suggest that the use of LipoMin reagent indeed retains substantial EV population as represented by CD81 protein.

Taken together, all available data support the efficacy of LipoMin in post-SEC, a second-step process to remove lipoproteins while substantially retaining EV. ELISA data (Figure 4g,h) suggest ApoA1 and ApoB for SEC + LipoMin were reduced to 0.85 and 1.79%, respectively, compared to the SEC-only process. This amount of ApoA1 and ApoB is comparable to that of the SEC + DG process, suggesting that LipoMin should have the potential to replace DG in its role of lipoprotein removal without sacrificing CD81 and flotillin proteins (Figure 4i,k). The EV purity data (Figure 4j) also support LipoMin’s potential toward high-purity EV isolation. Data using rEV (Figure S2) suggested that SEC + LipoMin recovered 46% of EV compared to SEC + ODG of 30%. Importantly, LipoMin with a roughly 10 min workflow should have a significant advantage over that of the time-consuming ultracentrifugation of DG (Figure 4l), implicating its potential in post-SEC workflow for high-purity EV isolation.

### 3.3 UC + LipoMin: LipoMin Added to UC-Processed Sample

Figure 6 presents the results of LipoMin reagent added to the resuspended pellet sample after differential ultracentrifugation. The rationale here is that the density of HDL overlaps significantly with that of EV and is compounded by the fact that HDLs are orders of magnitude more abundant than EV; thus, HDL is the major source of contamination in UC pellet.^15^ Further, it has been reported that >80% of studies used only UC for EV purification.^49^ Interrogation of LipoMin to further reduce lipoproteins from the UC pellet seems warranted.

Figure 6a illustrates the workflow of UC + LipoMin. After UC (first step), usage of LipoMin (second step) required: resuspend UC pellet, add LipoMin reagent, mix gently via pipetting, place magnet stand next to Eppendorf, and pipette out sample containing purified EV. Figure 6b,c presents TEM images for UC-only and UC + LipoMin processes; both wide-field and high-resolution images are shown.

Figure 6d,e presents ELISA results for LP markers ApoA1 and ApoB, respectively, for UC-only, UC + LipoMin 0.5× (half the volume of LipoMin), and UC + LipoMin (both at the same concentration). Results show that LipoMin reduces both ApoA1 and ApoB from stand-alone UC, with 23.5% of ApoA1 (76.5% reduction) and 0.22% ApoB (99.8% reduction) remaining. If further ApoA1 removal is desired, repeated use of LipoMin can be processed.

Figure 6f shows the CD81 ELISA data with two volumes of LipoMin. Results implicate that UC + LipoMin retained over 90% of the CD81 protein compared to the UC-only process. Thus, the removal of LP (Figure 6d,e) does not seem to comprise EV content. Although data on EV purity (similar to Figure 4j) cannot be directly calculated (due to CD81 protein concentrations being unavailable), Figure 6d–f clearly implicates that EV purity for UC + LipoMin would increase significantly over that of UC-only due to a pronounced reduction of combined ApoA1 and ApoB. The fold change (eq 2) in EV purity can be estimated by assuming that CD81 expression is much less than ApoA1 and ApoB combined (hence ignore CD81 contribution in the denominator of the equation in Figure 4j), i.e.
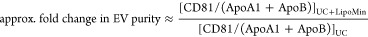
2and resulted in approximate fold change in EV purity of 6.57, which is considered substantial.

Figure 6g presents WB results for ApoB, ApoA1, flotillin, CD81, and albumin for various processes. UC-processed sample co-isolated a significant amount of ApoA1 (mainly HDL) with EV while less dense ApoB (LDL) can be effectively removed. Further removal of ApoB with LipoMin is attained. Removal of ApoA1 is also evident with efficacy increases with LipoMin reagent volume. Importantly, bonafide EV markers flotillin and CD81 show uncompromised expression compared to stand-alone UC. Reduction of albumin with LipoMin from the UC pellet is also supported. Taken together, WB results are in accordance with ELISA data (Figure 6d–f) in every aspect.

### 3.4 Colorectal Cancer (CRC) and Alzheimer’s Disease (AD) Samples

**Figure 7:**
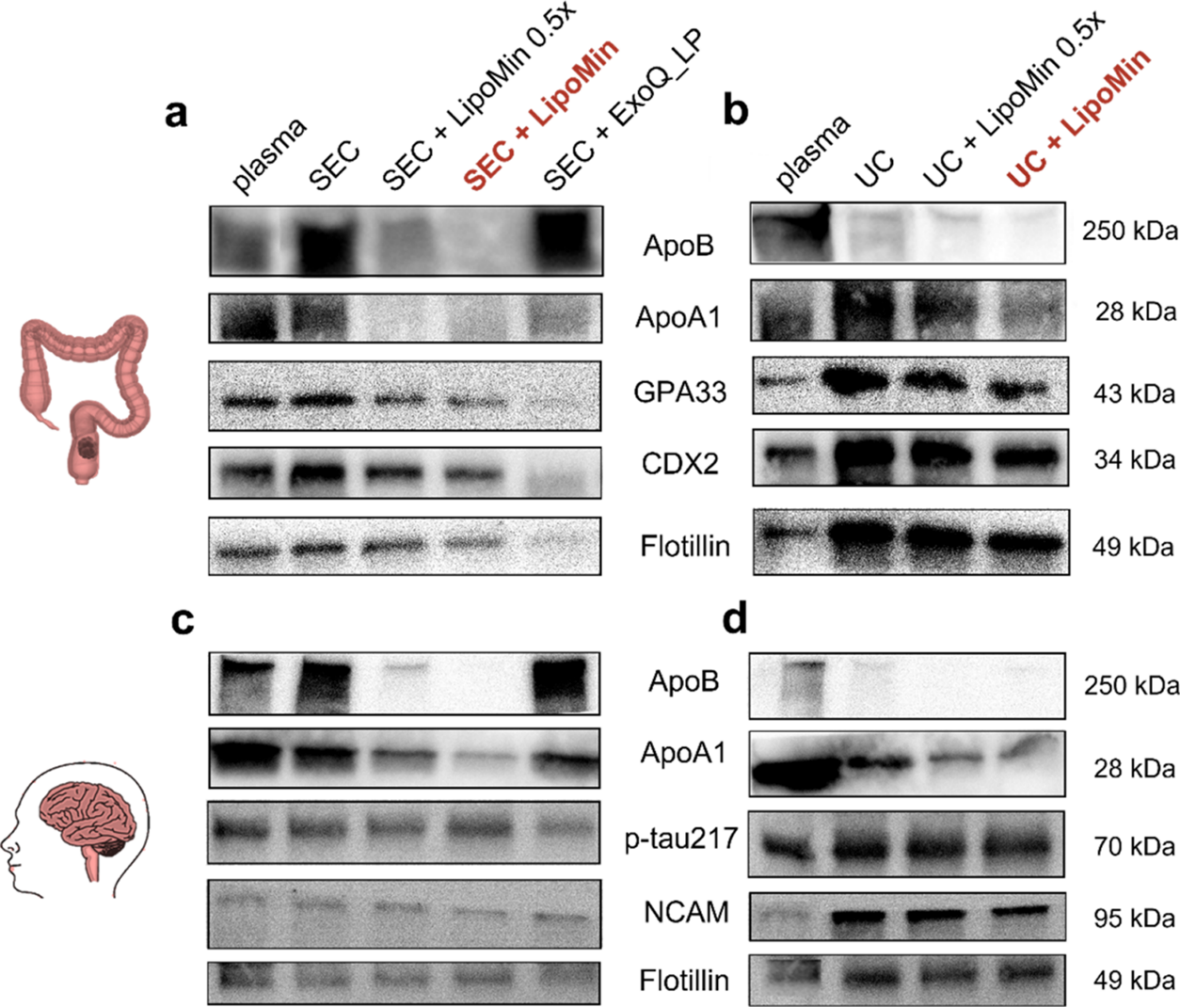
Western blot results of proteins related to colorectal cancer (CRC) (a, b) and Alzheimer’s disease (AD) (c, d). (a) CRC plasma processed by SEC, SEC + LipoMin 0.5× (half-volume as LipoMin), SEC + LipoMin, and SEC + ExoQ_LP. (b) CRC plasma processed by UC-related processes, same as (a) except without ExoQ_LP. (c) AD plasma processed by SEC-related processes the same as (a). (d) AD plasma processed by UC-related processes same as (b). CRC-relevant proteins tested include GAP33 and CDX2. AD-relevant proteins tested include phosphorylated tau at threonine 217 (p-tau217). Other proteins (ApoB, ApoA1, flotillin, NCAM) are also studied and provided for comparison. WB was performed twice to ensure consistency.

LipoMin was used to process the pathological samples. Figure 7 presents the results of WB for proteins related to colorectal cancer (CRC) and Alzheimer’s disease (AD). Plasma samples from patients with CRC and AD were processed with various methods, including UC as a stand-alone, UC followed by LipoMin, SEC as a stand-alone, SEC followed by LipoMin, and SEC followed by ExoQ_LP processes.

For CRC, Figure 7a,b, for SEC- and UC-processed samples, respectively, shows the overall efficacy of EV purification as a result of LipoMin. Overall, LP contents (ApoB and ApoA1) are reduced significantly, in agreement with earlier data of Figures 4k and 6g. Meanwhile, signals for EV (flotillin) and CRC markers (GPA33 and CDX2)^50^ are not comprised. Data from both SEC- and UC-processed with LipoMin seem to lend solid support for the reagent in the CRC setting.

For AD, Figure 7c,d, for SEC- and UC-processed samples, respectively, shows similar patterns as in corresponding Figure 7a,b for CRC. Figure 7c suggests that SEC + LipoMin also maintained a significant signal for EV (Flotillin) and AD markers (phosphorylated tau at threonine 217; p-tau217) and neuronal protein (NCAM).^51,52^Figure 7d shows UC and UC + LipoMin where UC + LipoMin maintained solid WB signals for p-tau217, NCAM, and Flotillin.

## 4 Discussion

Perhaps the most important aspect of high-purity isolation of EV from plasma is to balance this lofty goal with high efficiency of EV recovery, which is often a trade-off since the removal of lipoproteins would also simultaneously deplete some EV.^42^ In this work, for the SEC + LipoMin process, ELISA data (Figure 4g–i) showed ApoA1 decreased to 0.85% and ApoB decreased to 1.79%, compared to that of SEC EV pooled fractions, while CD81 decreased only 7.0%. One study (as a reviewer pointed out) purified rat blood using DG followed by bind-elute chromatography (BEC), among others, and resulted in albumin and LP contamination below the detection limit in EV-rich fractions.^37^ However, the protein in this EV-rich fraction was only 1.0% of that of the pre-BEC process (average of 295.3 μg eluted vs 28,400 μg input).^37^

Another point a reviewer made was that the LipoMin reagent is not as able to remove ApoA1 after UC (Figure 6d) as the removal of ApoA1 after SEC (Figure 4g). This is because, after SEC, most of the remaining ApoA1 are contained in chylomicrons (CM) due to its size (see Figure 1). Since CM contain both ApoA1 and ApoB, and LipoMin is effective in the removal of ApoB, CM are also removed with LipoMin usage, along with the removal of ApoA1 as a side benefit. For UC, however, ApoA1 mostly are in HDL. With HDL of approximately the same density as EV, HDL are also pelleted and LipoMin is not as effective in the removal of ApoA1 after UC (compared with the removal of ApoA1 after SEC).

EV purity for the SEC + LipoMin process may be further improved. Figure 4j shows that the concentration ratio (used in calculating the EV purity) for SEC was 2.52 × 10^–5^ and for SEC + LipoMin 1.45 × 10^–3^, resulted in a very attractive 57-fold increase in EV purity. However, with LP roughly 5 to 6 orders more than that of EV,^5^ a further increase in EV purity can perhaps be accomplished by repeated use of LipoMin or use greater volume of LipoMin. For example, another usage of LipoMin with the same volume may enable an additional 50-fold increase (rough estimate since fold change will decrease with repeated use of LipoMin), or a total of 2850-fold change (=57 × 50), or about 3 orders of magnitude LP reduction. The effect on EV should be acceptable since 93% of CD81 expression was retained after one LipoMin process (Figure 4i), or roughly 87% (=0.93^2^) after twice usage of LipoMin.

The use of rEV as a standard EV material provides an independent source to interrogate the efficacy of EV recovery by using LipoMin. Prof. Hendrix’s group used both fNTA and ELISA (p24, a subunit of the gag polyprotein) demonstrated convincingly that SEC followed by the ODG (OptiPrep density gradient) recovered around 30% of pre-ODG rEV. The authors emphasized that the orthogonal implementation of size and density-based separation of sample EV result in highly specific EV, implying minimal contamination. However, the high specificity of EV using second-step ODG comes at a rather steep cost in EV recovery. In contrast, CD81 ELISA data in Figure 5 of rEV spiked in SEC pooled fraction showed 89% of CD81 protein recovered after processing with LipoMin (also see Figure 4i of 93%). Importantly, Figure 4g,h shows that lipoprotein contaminants decreased to less than 1% for ApoA1 and less than 2% for ApoB, both comparable to that of DG. In short, data suggest that LipoMin as a second-step process recovers around 90% EV compared to 30% EV using DG, while decreasing the same amount of LP contaminants and saving 96% of processing time.

A reviewer pointed out that the use of LipoMin to remove LP from UC pellet as a second-step process should be compared to multiple cycles of UC in the removal of LP. Although this should be executed ideally, a study of EV enrichment from human serum using multiple cycles of centrifugation concluded that “five-cycle repetition of UC or centrifugation is necessary for successful removal of nonexosomal proteins in the enrichment of exosomes from human serum”.^53^ Clearly, five cycles of UC would gravely decrease process efficiency. Hence, we opted not to compare multiple UC cycles with those of UC followed by LipoMin. The UC + LipoMin process retained 23.5% of ApoA1 and 0.22% of ApoB of corresponding quantities from UC pellet (Figure 6d,e).

Western blot data in CRC and AD samples (Figure 7), although only covering two diseases, represent two distinct cases of pathogenesis. Notwithstanding these differences, regardless of SEC- and UC-processed samples and with or without LipoMin, all appear to provide clear protein signature for EV and disease biomarkers tested. The difference with and without LipoMin clearly lies in the amount of ApoA1 and ApoB removed, hence the significant difference in biomarker purity.

## 5 Conclusions

Extracellular vesicles in the circulation have been implicated to play a paramount role in inflammation, disease progression, and modulation of therapeutic response, among others. However, deep understanding of EV has been hampered by the fact that LP are phenomenally 5–6 orders of magnitude more than that of EV. This huge difference between LP and EV is being mitigated by two-step approaches in EV purification process, e.g., SEC (size) followed by density gradient (density) approaches studied. Nonetheless, the total workflow can take 6–18 h, substantially hindering EV purification workflow toward the realization of their utility.

This work provided data on the use of LipoMin reagent containing functionalized magnetic beads as a second-step, rapid (about 10 min), and straightforward methodology to remove a substantial amount of lipoproteins, particularly for SEC- and UC-processed samples toward high-purity EV. Results from sandwich ELISA for the SEC + LipoMin approach showed that only 1.79% ApoB remained (or 98.2% of ApoB removed) from post-SEC pooled EV fractions (Figure 4h). This is comparable to the current SEC + DG approach of 2.18% of ApoB remaining (Figure 4h). Equally important, the recovery rate of EV, as quantified by CD81, for the SEC + LipoMin approach remains high, at 93.0% (Figure 4i).

EV purity data (Figure 4j) suggest that the SEC + LipoMin process resulted in a fold change of 57.6-fold over a stand-alone SEC process. This is significantly higher than 3.7-fold change for SEC + DG, suggesting that LipoMin may be used to replace DG as a post-SEC, second-step process.

Pathological plasma samples from colorectal cancer (CRC) and Alzheimer’s disease (AD) patients were processed with SEC, SEC + LipoMin, SEC + ExoQ_LP, UC, and UC + LipoMin. These samples, with many confounding elements, enabled stringent interrogation of LipoMin in the clinical setting. Data showed the use of LipoMin removed substantial LP while retaining organ and disease-related markers and EV signatures: GPA33 (colon), CDX2 (CRC), NCAM (neuron), p-tau217 (AD), and flotillin (EV).

Taken together, data presented herein support the use of the LipoMin reagent as an effective second-step approach to further remove ApoA1 and ApoB from SEC- and UC-processed samples, rendering high-purity EV for subsequent analysis. With about 10 min workflow, LipoMin has the potential to enable EV isolation as a strong alternative to the widely used, second-step density gradient ultracentrifugation.

## Supporting Information Available

Antibodies used in this study (Table S1); LipoMin reagent workflow; removal of lipoproteins using ExoQuick-LP, a commercial kit; and EV-containing fractions in the SEC + DG process (PDF)
