# Supplementary material for: Improving the Purity of Extracellular Vesicles by Removal of Lipoproteins from Size Exclusion Chromatography- and Ultracentrifugation-Processed Samples Using Glycosaminoglycan-Functionalized Magnetic Beads
Source: ACS Appl Mater Interfaces. 2024 Aug 16;16(34):44386–98. doi: 10.1021/acsami.4c03869 (PMC11367580; doi:10.1021/acsami.4c03869)
Supplement: Supplementary file 1 — am4c03869_si_001.pdf [file am4c03869_si_001.pdf]

Supporting information

Improving the Purity of Extracellular Vesicles by Removal of Lipoproteins from Size Exclusion Chromatography- and Ultracentrifugation-Processed Samples Using Glycosaminoglycan-Functionalized Magnetic Beads

Cheng-Yu Chou[1,+], Po-Chieh Chiang[2,+], Chih-Chi Li[3], Jheng-Wun Chang[1], Po-Han Lu[1], Wei-Fan Hsu[1,2], Li-Chun Chang[4,5], Jung-Lung Hsu[6,7,8], Ming-Shiang Wu[4], and Andrew M. Wo[1,2*]

1 Institute of Applied Mechanics, National Taiwan University, Taipei 106319, Taiwan;

2 Reliance Biosciences, Inc., New Taipei City 231023, Taiwan;

3 Graduate Institute of Biomedical Electronics and Bioinformatics, National Taiwan University, Taipei 106319, Taiwan;

4 Department of Internal Medicine, National Taiwan University Hospital, Taipei 100225, Taiwan;

5 Health Management Center, National Taiwan University Hospital, Taipei 100225, Taiwan;

6 Department of Neurology, New Taipei Municipal TuCheng Hospital, New Taipei City, 236017, Taiwan;

7 Department of Neurology, Chang Gung Memorial Hospital Linkou Medical Center and College of Medicine, Neuroscience Research Center, Chang-Gung University, Linkou, Taoyuan, 33302, Taiwan;

8 Graduate Institute of Mind, Brain, & Consciousness, Taipei Medical University, Taipei, 110301, Taiwan.

+ Both authors contributed equally

*Corresponding author:

Andrew M. Wo, PhD
1 Roosevelt Road, Sec. 4
Institute of Applied Mechanics
National Taiwan University
Taipei 106319, Taiwan.
Phone: +8862-3366-5656. Fax: +8862-2363-9290.
Email: andrew@iam.ntu.edu.tw

EXPERIMENTAL SECTION

Table S1 Antibodies used in this study

Antibody	Company	Clone	Dilution ratio
Goat polyclonal biotin anti-ApoB	abcam, UK	-	1:1,600 (ELISA)<br>1:10,000 (WB)
Mouse monoclonal anti-ApoB	Novus, USA	7B8	1:10,000 (ELISA)
Goat polyclonal biotin anti-ApoA1	abcam, UK	-	1:800 (ELISA)<br>1:5,000 (WB)
Mouse monoclonal anti-ApoA1	abcam, UK	12C8	1:5,000 (ELISA)
Mouse monoclonal anti-CD81	Biolegend, USA	5A6	1:200 (ELISA)
Rabbit polyclonal anti-CD81	ThermoFisher, USA	-	1:1,000 (ELISA)
Mouse monoclonal biotin anti-CD81	Novus, USA	1D6	1:1,000 (WB)
Rat monoclonal anti-flotillin 1 HRP	Biolegend, USA	W16108A	1:1,000 (WB)
Rat monoclonal anti-human A33	R&D system, USA	402104	1:1,000 (WB)
Rabbit monoclonal anti-CDX2	abcam, UK	EPR2764Y	1:1,000 (WB)
Rabbit monoclonal anti-p-tau217	Reliance Biosciences, Taiwan	PSL263	1:1,000 (WB)
Mouse monoclonal anti-NCAM	Leadgene, Taiwan	MEM-188	1:1,000 (WB)
Mouse monoclonal anti-Albumin	abcam, UK	15C7	1:2000 (WB)
Goat anti-mouse IgG HRP	Biolegend, USA	-	1:100,000 (ELISA)<br>1:10,000 (WB)
Goat anti-mouse IgG1 HRP	ThermoFisher, USA	-	1:20,000 (ELISA)<br>1:10,000 (WB)
Goat anti-rabbit IgG HRP	Leadgene, Taiwan	-	1:20,000 (ELISA)<br>1:10,000 (WB)
Streptavidin-HRP solution	abcam, UK	-	1:10,000 (WB)

LipoMin Reagent Workflow

Sample preparation. LipoMin (product# RB02001, Reliance Biosciences Inc., Taiwan) usage allows flexibility in sample preparation. The tables below were used to determine the amounts of sample and LipoMin reagent, provided by the manufacturer. For SEC pooled EV fractions, 5 µl was pipetted out and diluted with 95 µl PBS to 100 µl total volume, resulting in a 20x dilution of the SEC sample. For resuspended UC pellet, 10 µl of sample was pipetted out and diluted with 90 µl PBS to 100 µl total volume, resulting in a 10x dilution of UC sample. One can adjust sample volume and LipoMin reagent proportionately.

LipoMin preparation. LipoMin reagent only required mixing with buffer (provided) and a 10-minute incubation (e.g. on rotating mixer) before use. The table below provided suggested portion of LipoMin for corresponding sample volume.

SEC sample	LipoMin 0.5x	LipoMin
5 µl	20 µl	40 µl
20 µl	80 µl	160 µl

UC sample	LipoMin 0.5x	LipoMin
10 µl	5 µl	10 µl
25 µl	12.5 µl	25 µl

Notes: (1) "LipoMin 0.5x" is half the volume of LipoMin", both with the same concentration. Additional LipoMin can also be used to further remove LP if needed. (2) If the sample has not previous treated with SEC, UC, or other means, substantially more LipoMin volume will be needed. (3) Adjustment of LipoMin volume can also be made proportionally.

LipoMin usage for removal of lipoprotein. To remove lipoproteins, a magnetic separator was placed next to the sample mixed with LipoMin. The purified sample, containing EV with lipoproteins removed, was transferred into a clean tube for subsequent processing.

Removal of Lipoproteins using ExoQuick-LP - a Commercial Kit

ExoQuick-LP kit (System Biosciences, CA, USA) was used to further remove LP from SEC EV pooled fractions enabling extremely pure EVs. The manufacturer's instructions were followed to wash and mix the pre-clearing-reagent with the magnetic beads. These functionalized magnetic beads may bind with LP antibodies due to the antibody affinity for LP removal.

The sample was mixed with ExoQuick-LP magnetic beads using the recommended volume from manufacturer's instructions. After thorough mixing by pipetting, the mixture was incubated on a rotator for 3 hours at 4°C. Subsequently, a magnet stand was used to remove ExoQuick-LP magnetic beads capturing LP from the mixture. Supernatant containing purified EV was transferred to a new container for downstream processing.

EV-Containing Fractions in the SEC+DG Process

The SEC+DG processed sample was handled in the following manner prior to comparison with SEC+LipoMin processed sample. Post-SEC EV-containing fractions F3 to F5 were pooled, 2-fold diluted, and loaded to the sucrose density gradient (DG). After centrifugation, sequential ten 1 mL fractions were collected and quantified using CD81 sandwich ELISA. Figure S1 ELISA data revealed that F8 and F9 have the highest CD81 protein concentration. Thus, SEC+DG processed F8 and F9 were pooled for comparison with SEC+LipoMin.

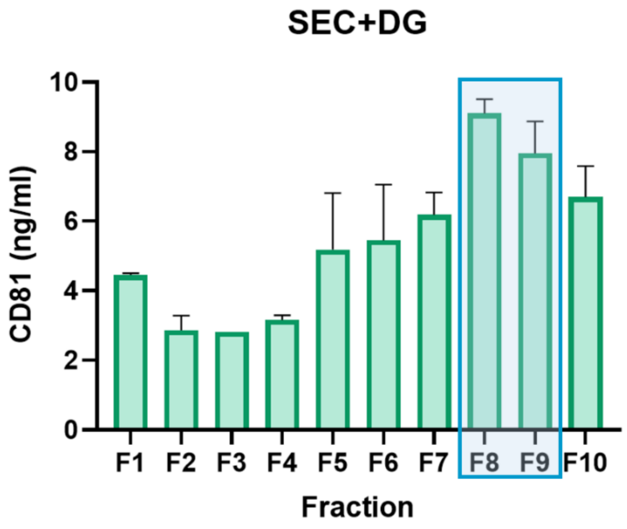

Figure S1. CD81 sandwich ELISA analysis of SEC followed by DG treatment of plasma sample. Data showed that F8 and F9 had the highest CD81 protein concentration. Thus, SEC+DG processed F8 and F9 were pooled for comparison with SEC+LipoMin.

Efficiency of EV Recovery using Recombinant EV (rEV) as Reference Material

In an important milestone in standardization and comparison of EV data, recombinant EV (rEV) with fluorescence was generated as a biological reference material by Prof. Hendrix's group [1]. Thus, spiking rEV in pre-processed samples enables direct comparison of efficiency of EV recovery for various EV isolation methods. For detail discussion, see Section 2.7 and discussion in the second to the last paragraph in Section 3.2.

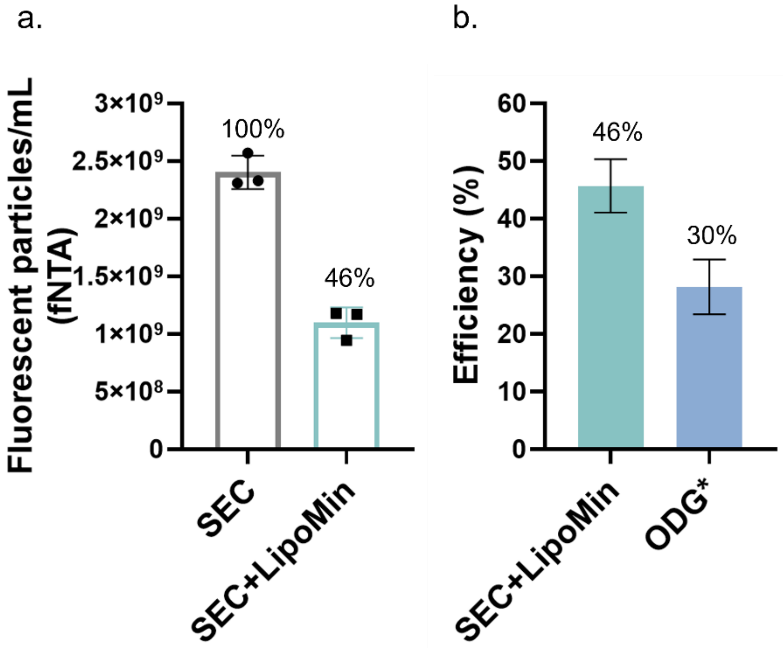

Figure S2. Use of recombinant EV (rEV) as reference material to calculate the efficiency of EV recovery for LipoMin processing, and comparing with Optiprep DG (ODG) [1]. (a) Concentrations of rEV spiked in SEC-only and SEC+LipoMin sample directly quantified by fluorescent NTA (fNTA). (b) Calculation of efficiencies of EV recovery from SEC+LipoMin and ODG samples. (* This ODG data points were taken from published work by Prof. Hendrix's group [1], or ref. 41 in the paper).

Reference in SI:

(1) Geeurickx, E.; Tulkens, J.; Dhondt, B.; Van Deun, J.; Lippens, L.; Vergauwen, G.; Heyrman, E.; De Sutter, D.; Gevaert, K.; Impens, F.; et al. The generation and use of recombinant extracellular vesicles as biological reference material. *Nat Commun* **2019**, *10* (1), 3288. DOI: 10.1038/s41467-019-11182-0.
